# Circadian Rhythm Dysfunction Accelerates Disease Progression in a Mouse Model With Amyotrophic Lateral Sclerosis

**DOI:** 10.3389/fneur.2018.00218

**Published:** 2018-04-24

**Authors:** Zhilin Huang, Qiang Liu, Yu Peng, Jiaying Dai, Youna Xie, Weineng Chen, Simei Long, Zhong Pei, Huanxing Su, Xiaoli Yao

**Affiliations:** ^1^Department of Neurology, National Key Clinical Department and Key Discipline of Neurology, Guangdong Key Clinical Laboratory for Diagnosis and Treatment of Major Neurological Diseases, The First Affiliated Hospital, Sun Yat-sen University, Guangzhou, China; ^2^State Key Laboratory of Quality Research in Chinese Medicine, Institute of Chinese Medical Sciences, University of Macau, Macao, China; ^3^Comprehensive Department, Sun Yat-sen Memorial Hospital affiliated to Sun Yat-sen University, Guangzhou, China

**Keywords:** circadian rhythm dysfunction, amyotrophic lateral sclerosis, inflammation, NF-κB, cyanobacteria

## Abstract

Amyotrophic lateral sclerosis (ALS) is a fatal neurodegenerative disease caused by interactions between environmental factors and genetic susceptibility. Circadian rhythm dysfunction (CRD) is a significant contributor to neurodegenerative conditions such as Alzheimer’s disease and Parkinson’s disease. However, whether CRD contributes to the progression of ALS remains little known. We performed behavioral and physiological tests on SOD1G93A ALS model mice with and without artificially induced CRD, and on wild-type controls; we also analyzed spinal cord samples histologically for differences between groups. We found that CRD accelerated the disease onset and progression of ALS in model mice, as demonstrated by aggravated functional deficits and weight loss, as well as increased motor neuron loss, activated gliosis, and nuclear factor κB-mediated inflammation in the spinal cord. We also found an increasing abundance of enteric cyanobacteria in the ALS model mice shortly after disease onset that was further enhanced by CRD. Our study provides initial evidence on the CRD as a risk factor for ALS, and intestinal cyanobacteria may be involved.

## Introduction

Amyotrophic lateral sclerosis (ALS) is a fatal neurodegenerative disease that impairs motor neurons (MNs) in the brain and spinal cord, normally causing death within 3–5 years of onset (1). Although the precise mechanisms remain unclear, the consensus is that ALS is the pathological outcome of a combination of genetic and environmental factors. So far, more than 180 potential genetic risk factors have been identified, including mutations in SOD1 (Cu/Zn superoxide dismutase 1), TDP-43 (TAR DNA-binding protein 43), FUS (fused in sarcoma/translated in liposarcoma), and an increased number of repeats in C9ORF72 (2). Numerous studies have sought to identify ALS-related environmental factors, such as excessive exercise, mercury and heavy metal toxicity, geographical clustering, electromagnetic fields, head injuries, and neurotoxins (3, 4). Specifically, sleep disorders including nocturnal hypoventilation, restless legs syndrome, mood disorders, sleep-disordered breathing, and circadian disturbances occur frequently in ALS patients (5, 6). Some studies suggest that circadian rhythm dysfunction (CRD) alters molecular, cellular, or physiologic functions that favor the development of neurodegenerative conditions (7–9).

The circadian system is considered to be a complex network of positive and negative feedback loops consisting of clock genes (clock and bmal1; per1-3 and cry1-2) and their transcriptional products (BMAL1-CLOCK complexes; PER1-3 and CRY1-2) and regulated by posttranslational events, such as phosphorylation and dephosphorylation (10, 11). The suprachiasmatic nucleus (SCN) is the core circadian clock, and is entrained by a zeitgeber (“time giver”), a signal such as the light–dark (diurnal) cycle transduced through the retinohypothalamic tract (12). Studies have shown that peripheral cells and tissues also have their own rhythmicity in the immune (13), cardiovascular (14), and digestive systems (15) with time. The SCN can synchronize both central nervous system rhythms and those of peripheral tissues through neural and humoral controls (16). Accumulating evidence demonstrates that CRD contributes to the progression of neurodegenerative diseases such as Alzheimer’s disease (AD) and Parkinson’s disease (PD) (7–9, 17, 18). Several mechanisms of functional impairment by abnormal circadian clocks have been proposed, including epigenetic signals, cellular metabolic changes, and inflammation (19). Gliosis play crucial roles in MN death *via* non-cell-autonomous mechanisms (20). A large body of evidence indicates that nuclear factor κB (NF-κB)-mediated signaling plays an important role in neuronal survival in many pathological conditions [for a review see Ref. (21)]. The p65 subunit is considered the most important NF-κB subunit in the nervous system, and the activation of NF-κB has been shown to involve the phosphorylation of p65 (p-p65) and IκB kinase (p-IKK) (22).

The gut and its microbiome are also regulated by the SCN circadian clock in a complex way. Although the digestive system cannot perceive illumination, the SCN, driven by the light zeitgeber, regulates digestive rhythms in a distinct way, by affecting the intestinal bacterial compositions of the host (23). However, other signals, such as feeding and drinking, were also found to act as zeitgebers for digestive clocks. Thus, it is quite possible for an enteric clock to be desynchronized from the SCN clock rhythm, an event that can result in broad-spectrum disorders like metabolic diseases (24). Indeed, a recent study has stated that the intestinal microbiome can regulate host transcriptomic oscillations through regulating rhythmic biogeography and the metabolome (25).

Increasing evidence suggests that disturbances in the gut microbiota contribute to the development of neurological diseases. For example, colonization with microbiota from patients with PD enhances motor impairments in animal models of PD (26). It has also been hypothesized that an unknown toxin produced by gut microbes causes ALS through the gut–microbe–brain axis (27, 28). Cyanobacteria (formerly known as blue-green algae) are common in water, cycads, and intestinal flora. Epidemiological studies suggest that the areas near rivers and lakes with cyanobacteria blooms, including Wisconsin, New Hampshire, and Southern France, had a higher incidence of ALS (29). Interestingly, cyanobacteria possess an internal clock system with a three-protein (Kai A/B/C) oscillator that maintains rhythmicity in certain biological activities, such as nitrogen fixation. Kai B folds into two different three-dimensional structures that can drive the internal transition between day and night (30). In addition, nitrogen starvation of cyanobacteria can trigger the production of β-*N*-methylamino-l-alanine (BMAA), a neurotoxin which inhibits MN growth (31). Very little information on the relationship between circadian rhythms, cyanobacteria, and ALS progression is available, particularly regarding alterations in gut microbiome compositions and the abundance of cyanobacteria at different stages in the progression of ALS. Therefore, the present study investigated whether CRD could alter either cyanobacteria abundance in gut microbiome compositions or disease progression in adult ALS model mice, or both.

## Materials and Methods

### Animals and Circadian Dysfunction

Transgenic mice overexpressing human SOD1 were obtained from the Nanjing Biomedical Research Institute of Nanjing University, and the genotypes of their offspring were identified by polymerase chain reaction (PCR) using a standard protocol (32). Male transgenic [B6SJL-Tg (SOD1-G93A) 1GurJ] mice and their wild-type (WT) littermates were randomly divided into four groups: a SOD1G93A group with a light-induced CRD (ALS + CRD, *n* = 19), a SOD1G93A group with a normal light/dark cycle (ALS, *n* = 18), a WT group with a light-induced CRD (WT + CRD, *n* = 20), and a WT group with a normal light/dark cycle (WT, *n* = 15). All of the animals in CRD (altered rhythm) groups lived under a 20/4-h light/dark cycle from the age of 42 days until they were sacrificed; the animals in the other two groups lived under a normal 12/12-h light/dark cycle. Mice were sacrificed at the ages of 60, 90, or 120 days. All animals were provided *ad libitum* access to food and water in a temperature (25°C) and humidity (45–55%) -controlled environment. All experiments were performed according to the policies for the care and use of experimental animals and were approved by the Animal Ethical Committee at Sun Yat-Sen University, China. All animals were used only for one procedure and were humanely sacrificed under anesthesia after the completion of the experiment.

### Body Weight and Motor Function Assessments

The mice were transferred to the new light/dark environments and adapted for 1 week before measurement of body weight and motor function. Body weight and motor function measurements were performed every 3 days from the age of 50 days to one of the above three time points by a trained observer blinded to the experimental conditions. The final time point was set at the age of 120 days because ALS mice begin to die at this age. Using the widely accepted 4-point evaluation system for motor deficits in SOD1G93A mice (33), we defined the day of disease onset as the day on which hind limb tremors were evident when suspending the mouse in the air by its tail. Impending terminal impairment was defined as the day on which a mouse was unable to right itself within 30 s, and survival rate was calculated as a percentage of mice that had not reached the terminal stage.

### Fecal Bacteria Analysis

Gene sequencing of 16S ribosomal RNA (rRNA) was performed to characterize the distal gut microbiota (22,800 ± 35,017 trimmed sequences per sample). At each of the three time points, fresh fecal samples were collected in sterile 1.5-ml Eppendorf tubes at approximately 0900–1100 and immediately frozen in liquid nitrogen for 5 min. The fresh fecal samples were then transferred to −80°C until further processing. Microbial genomic DNA was extracted from stools, and the V4 region of the 16S rRNA gene was PCR-amplified using the following primers: 515F, GTGCCAGCMGCCGCGGTAA, and 806R, GGACTACHVGGGTWTCTAAT. The PCR products were sequenced on an Illumina Hiseq 2 × 300 bp platform (TinyGene Bio-Tech Co. Ltd.; Shanghai, China). The comparisons of relative abundance of bacterial taxa were performed based on the total number of classified reads for each sample.

### Immunolabeling

At the end of each time point, the mice were deeply anesthetized and transcardially perfused with ice-cold normal saline and followed by 4% paraformaldehyde. Then the spinal cords were carefully extracted, fixed in 4% paraformaldehyde for 24 h, and successively cryoprotected by immersion in 20 and 30% sucrose overnight. The cervical and lumbar enlargements were cut into 20-µm sections on a cryostat. Selected slices were blocked with 10% normal donkey serum (Beyotime) for 1 h at room temperature, and then incubated with primary antibodies to detect MNs (anti-choline acetyltransferase [ChAT], 1:400, OmnimAbs), astrocytes [anti-glial fibrillary acidic protein (GFAP), 1:400, Sigma], and microglia [anti-ionized calcium binding adaptor molecule 1 (Iba1), 1:200, Millipore] overnight at 4°C. Species-specific secondary antibodies were then added to incubate the slices at 37°C for 1 h in the dark. Images were captured using a Leica SP5 confocal microscope. The anterior horns of labeled sections were visualized and imaged with a laser scanning confocal microscope (Leica TCS SP5MP).

### Western Blotting

Mice were deeply anesthetized and transcardially perfused with ice-cold normal saline and the spinal cords were carefully removed. Total protein was extracted from each sample using the extraction protocol (Pierce). The extraction buffer contained protease and phosphatase inhibitors. Equal amounts of protein (10 µg) were loaded onto 8 or 10% sodium dodecyl sulfate polyacrylamide gel electrophoresis gel (SDS-PAGE) and separated. The proteins were transferred onto 0.22-µm polyvinylidene difluoride membranes (Millipore). Non-specific binding was blocked using 5% bovine serum albumin for 1 h at room temperature. The blots were subsequently incubated overnight at 4°C in primary antibodies with mild agitation. The primary antibodies used were rabbit anti-ChAT (1:1,000, OmnimAbs), rabbit anti-GFAP (1:1,000, Sigma), rabbit anti-Iba1 (1:1,000, Millipore), rabbit anti-p65 (1:1,000, CST), rabbit anti-p-p65 (1:1,000, CST), rabbit anti-p-Ikkα/β (1:1,000, CST), rabbit anti-β-actin (1:2,000, Sigma), and rabbit anti-GAPDH (1:2,000, Sigma). The membranes were washed three times for 10 min with Tris-buffered saline containing Tween-20 (TBS/T) and incubated with goat anti-rabbit secondary antibody (1:2,000, abcam) for 1 h at room temperature. The membranes were subsequently washed four times for 10 min with TBS/T. Immunoreactive proteins were visualized using the chemiluminescent horseradish peroxidase substrate (Pierce) and scanned using image analyzer software (Bio-Rad, Image lab 4.1).

### Analysis of MN Survival and Inflammation Activation in the Spinal Cord

Previous reports indicate that MNs in the cervical and lumbar segments of ALS model mice have very similar characteristics. We, therefore, studied sections from the cervical segment as representative of the spinal cord in the ALS model mice. About five sections per mice were labeled, and five fields of the ventral horn per slice were randomly selected at 250× magnification. We counted the numbers of ChAT-positive cells in these sections to assess the survival of MNs in the cervical spinal cord. We only counted ChAT-expressing cells clearly displaying a nucleolus located in the ventral horns of spinal sections. The number of surviving ventral horn MNs was described quantitatively as a percentage of the number counted in the WT controls. The luminescence intensity values of western blot bands were calculated by integrating the signals using ImageJ software. The normalized value of p-p65 is represented by the ratio of p65. All expression levels of gliosis and NF-κB are shown as rates of values in the WT controls.

### Statistical Analysis

All the data are presented as the means ± SEM. The comparisons of the relative abundances of microflora were analyzed using the Differentially Abundant Features program (Metastats, http://metastats.cbcb.umd.edu). A one-way of analysis of variance followed by Bonferroni’s *post hoc* test for multiple comparisons was used to analyze MN survival and glial activation. The comparisons between two groups were made using GraphPad Prism 5 (GraphPad Prism; La Jolla, CA, USA). *p*-Values < 0.05 were considered significant.

## Results

### CRD Accelerated Disease Onset and Progression in ALS Model Mice

**Figure 1:**
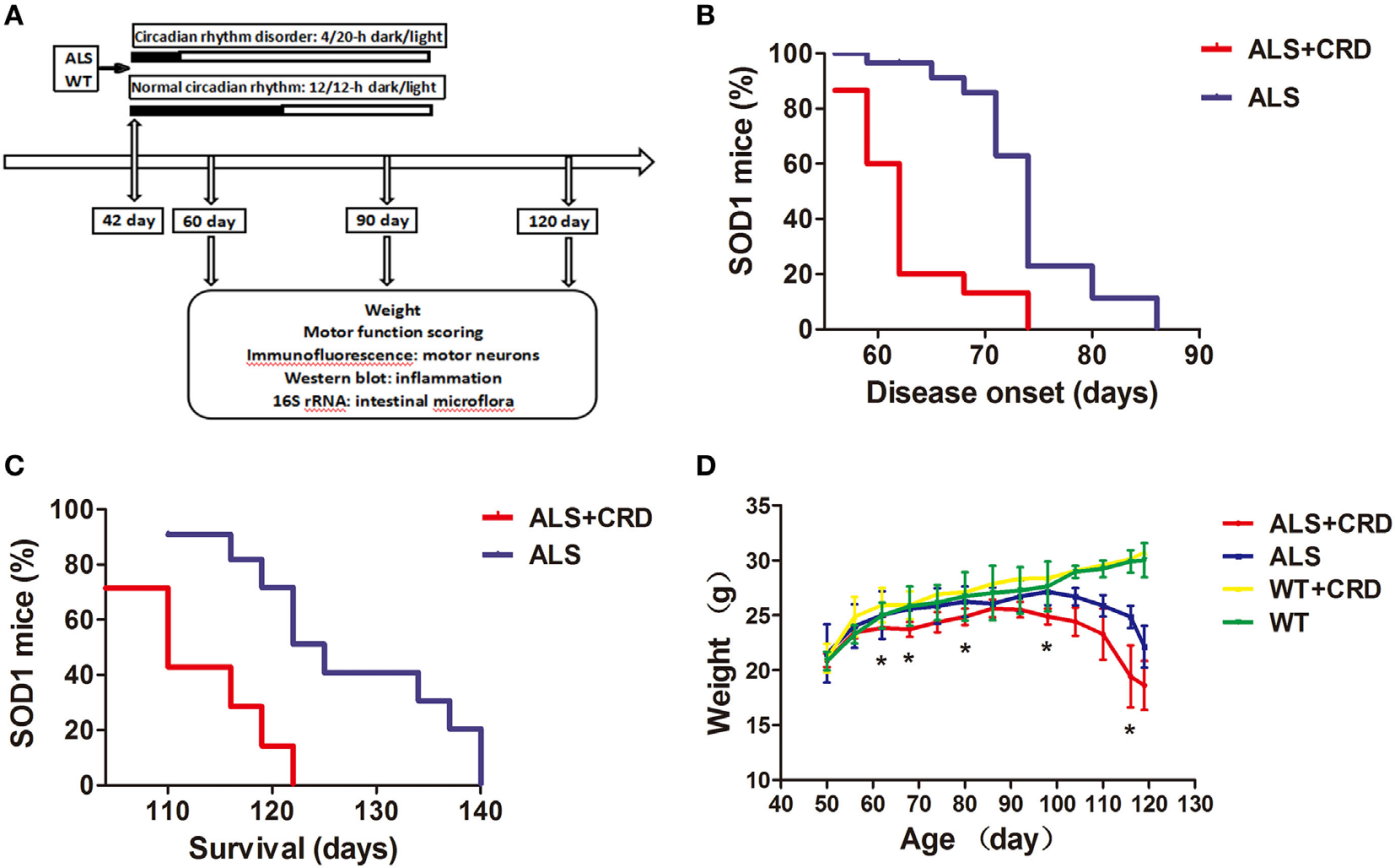
Circadian rhythm dysfunction (CRD) accelerated disease onset and progression in *SOD1G93A* mice. **(A)** A schematic showing the experimental procedures. **(B,C)** Survival curves: disease onsets and disease progression in the ALS + CRD and the ALS group. **(D)** Body weights loss of four groups starting from the day 50 to the day 119. *N* = 4–6 animals per group, *p < 0.05. Error bars represent SEM.

As shown in Figure 1A, the 42-day-old ALS model and WT mice were housed in a 20/4-h light/dark (ALS + CRD and WT + CRD groups) and a regular 12/12-h light/dark cycle (ALS and WT groups). Body weight and behavioral assessments were acquired every 3 days until euthanasia at days 60, 90, and 120. Symptom onset, as assessed based on motor function tests, occurred at 74.00 ± 2.60 days of age in the ALS group (*n* = 14). The end stage occurred at 130.00 ± 3.662 days of age in the ALS model mice (*n* = 7). In contrast, symptom onset was significantly advanced in the ALS + CRD group, occurring 12 days earlier than in the ALS group (Figure 1B; ALS + CRD: 62.00 ± 0.78 days of age, *n* = 15, *p* < 0.01); CRD also significantly shortened the lifespan of ALS mice (Figure 1C; ALS + CRD: 116.00 ± 3.00 days of age, *n* = 7, *p* < 0.05). We measured the change in body weight of the mice over time because body weight is a reliable index of disease progression in ALS (33). All mice in both the ALS and ALS + CRD groups demonstrated weight loss over time. A rapid and significant drop in body weight was found in the ALS group at the age of 83 days compared to the WT group (Figure 1D, *p* < 0.05). Notably, CRD accelerated the body weight loss of the ALS model mice such that the weight loss in the ALS + CRD group was significantly worse than that in the ALS group beginning at about 62 days of age (Figure 1D, *p* < 0.05). In contrast, WT mice gained weight during the experimental period; CRD did not affect body weight in the WT mice (Figure 1D, *p* > 0.05). Taken together, these results suggested that CRD accelerated the onset and progression of the ALS in the model mice.

### CRD Aggravated Neurodegeneration and Induced Inflammation in SOD1G93A Mice

**Figure 2:**
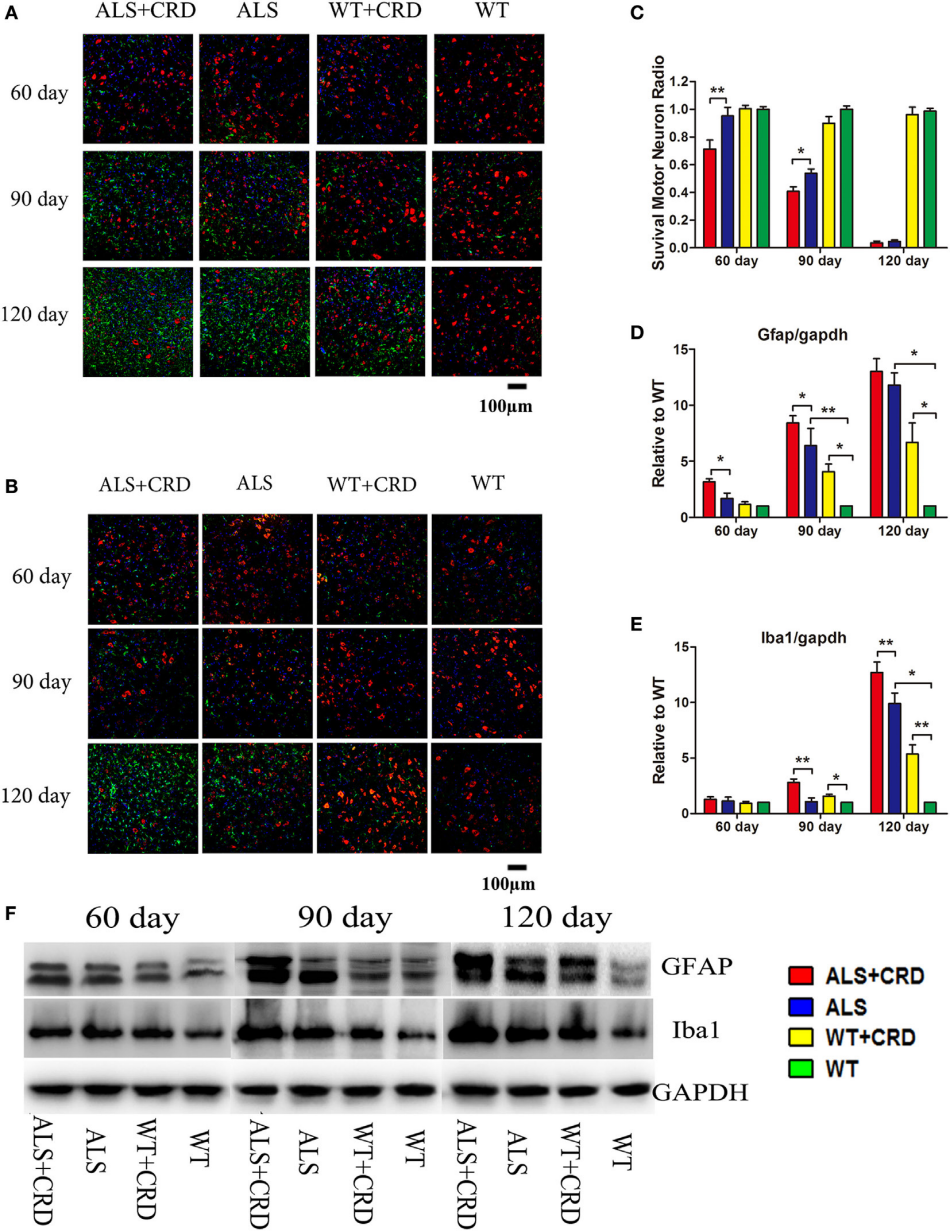
Circadian rhythm dysfunction (CRD) aggravated motor neuron (MN) loss and activated glial cells in *SOD1G93A* mice. **(A)** MNs [choline acetyltransferase (ChAT)-positive cells, red] and astrocytes [glial fibrillary acidic protein (GFAP)-positive cells, green] expression in four groups for three stages. **(B)** MNs (ChAT-positive cells, red) and microcytes (Iba1-positive cells, green) expression in four groups for three stages. Scale bars represent 100 µm. **(C)** Statistical evaluation of surviving MNs radio by counting the number of ChAT-positive cells. **p < 0.01; * *p <* 0.05 (one-way ANOVA, *n* = 4–6 animals per group). **(D,E)** GFAP and Iba1 expression was quantified at three stages by western blotting (one-way ANOVA, *n* = 2–3 animals per group). **(F)** Expression of GFAP and Iba1 in the spinal cord. **p < 0.01; *p < 0.05. Error bars represent SEM.

**Figure 3:**
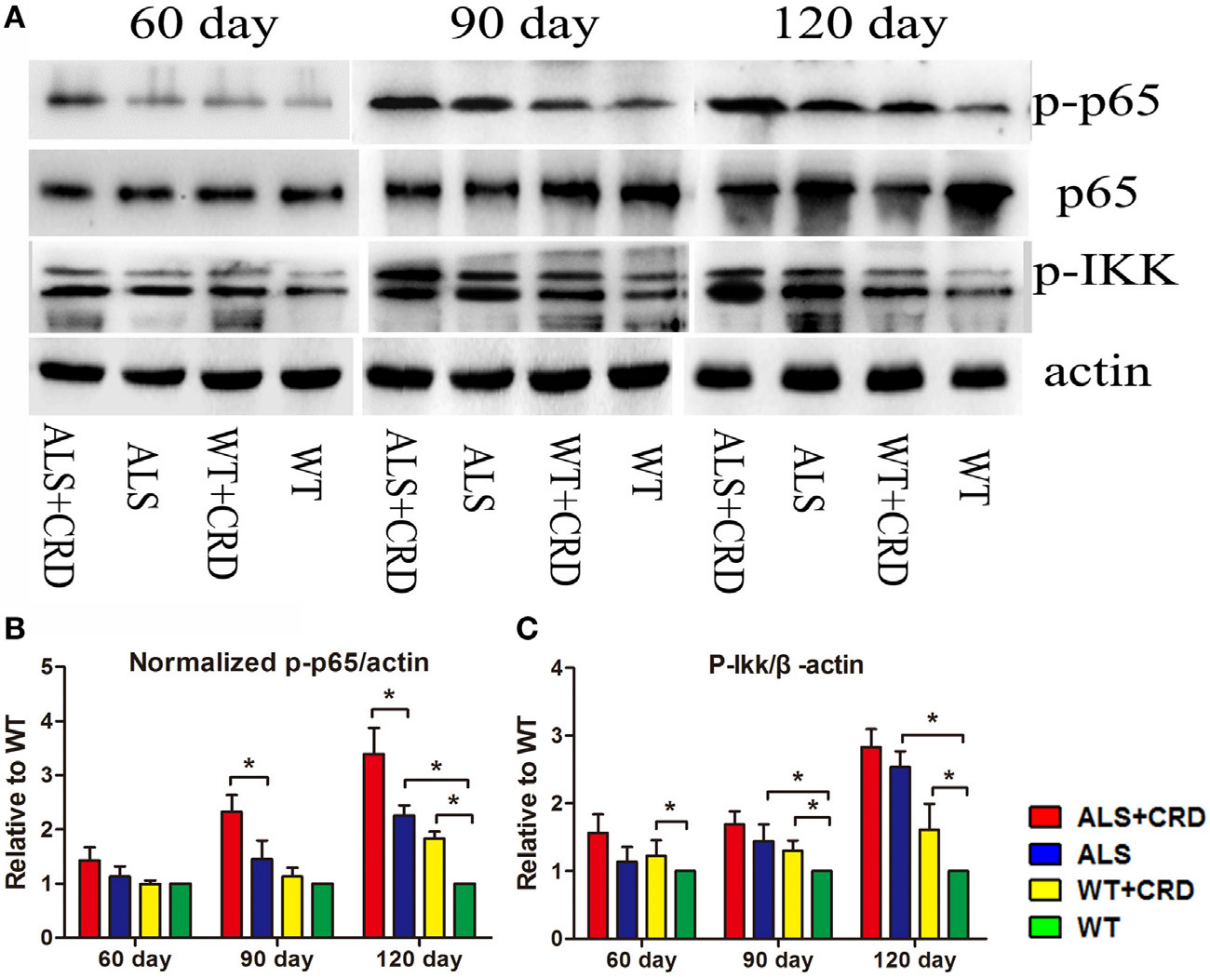
Circadian rhythm dysfunction (CRD) aggravated nuclear factor κB-mediated inflammation in *SOD1G93A* mice. **(A)** Expression of p65, p-p65, and p-IKK in the spinal cord. **(B,C)** Expression of p-IKK and normalized p-p65 was quantified at three stages by western blotting (one-way ANOVA, *n* = 2–3 animals per group). **p < 0.01; *p < 0.05. Error bars represent SEM.

The most typical pathological feature of ALS is the degeneration of spinal MNs. As shown in Figure 2A, there were no significant differences in the numbers of ChAT-positive cells at any time point between the WT + CRD group and WT groups, suggesting that chronic CRD did not lead to MN death in WT mice. In comparison, the percentage of remaining ChAT-positive cells in the ALS + CRD group was significantly lower than that of the ALS group at the 60-day time point (Figures 2A–C; *p* < 0.01) and the 90-day time point (Figures 2A–C; *p* < 0.05); the difference between the two groups at the 120-day time point was not significant because of the paucity of cells remaining in both the ALS + CRD and ALS groups at that time. Glial activation was assessed using both immunofluorescence and western blot measurements of GFAP and Iba1. Significantly more intense GFAP expression was detected in the ALS + CRD group relative to the ALS group at the 60- and 90-day time points (Figures 2A,D,F; *p* < 0.05). There were similar differences in Iba1 intensity between the ALS + CRD and ALS groups, although these differences were significant only at the 90-day and 120-day time points (Figures 2B,E,F; *p* < 0.01). The mice in the WT + CRD group also had a slight increase in GFAP and Iba1 expression when compared to those in the WT group. We measured the expression levels of p65, p-p65, and p-IKK, which are important constituents of the classical NF-κB inflammatory pathway. The expression levels of p-p65 and p-IKK were all slight increased in the ALS + CRD group when compared to the ALS group at each time point, but only had significance in p-p65 at days 90 and 120. A increase trend was observed in the WT + CRD group when compared to the WT group (Figures 3A–C, *p* < 0.05). All of the inflammatory markers assessed in the ALS group were increased significantly in association with disease progression. These data suggest that CRD exacerbated MNs loss and increased the numbers of gliosis and NF-κB inflammatory responses in the spinal anterior horn in transgenic ALS mice.

### CRD Increased Enteric Cyanobacteria Abundance in SOD1G93A Mice

**Figure 4:**
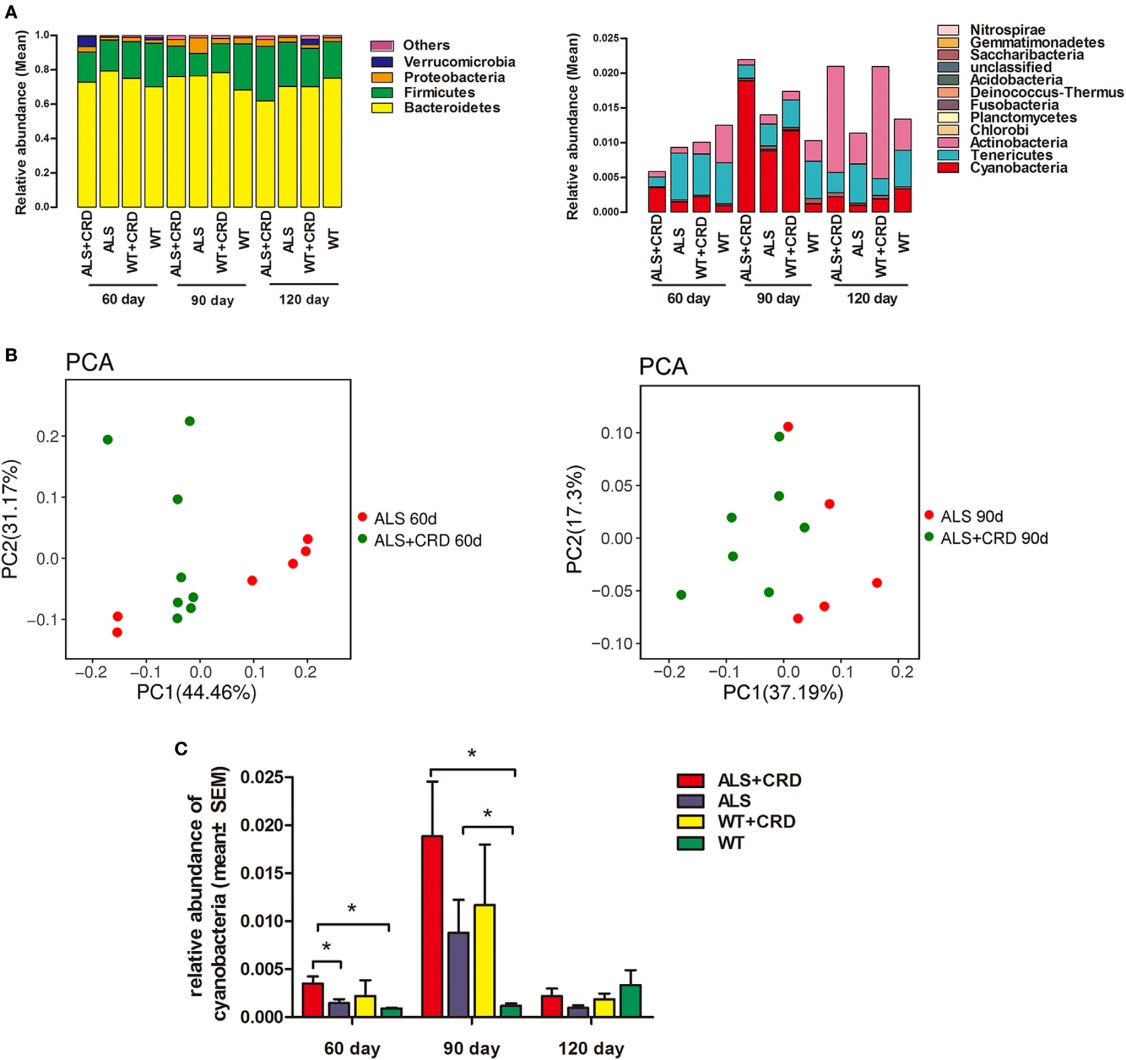
Circadian rhythm dysfunction (CRD) increased enteric cyanobacteria abundance in *SOD1G93A* mice. **(A)** Intestinal bacterial abundance profiles at the level of phylum among the four groups at three time points using 16S high throughput sequencing. Flora were presented in the form of averages. Sample capacity of each group was 4–8. **(B)** Differences and distances of each sample between the amyotrophic lateral sclerosis (ALS) + CRD group and the ALS group at day 60 and 90, respectively, reflected by Principle Component Analysis (PCA). **(C)** Comparisons of relative abundances of cyanobacteria among the four groups at different disease stages. *N* = 4–6 per group per time point, *p < 0.05. Error bars represent SEM.

Ribosomal RNA analyses allowed for a phylum-level description of the intestinal flora in the different experimental groups. The microbial gut compositions of the four groups at the three time points are presented in Figure 4A. The most abundant fecal microbes found in all four groups at all time points belonged to the phyla Bacteroidetes, Firmicutes, Proteobacteria, Verrucomicrobia, Deferribacteres, and Cyanobacteria (Figure 4A). Principle component analysis identified evident cluster differences in the compositions of gut flora between the ALS + CRD and ALS groups at both the 60- and 90-day time points (Figure 4B). Because epidemiological studies suggest that an increased abundance of cyanobacteria is associated with a higher incidence of ALS (29). Therefore, we focused our attention on the possible relationship between CRD and the abundance of enteric cyanobacteria. A significant increase in the relative quantity of cyanobacteria in the ALS model mice vs. the WT mice was found at the 90-day time point (Figure 4C, 0.8807 ± 0.3429% reads in the ALS group vs. 0.1180 ± 0.0264% reads in the WT group, *p* < 0.05). CRD significantly increased the cyanobacteria abundance beyond that of the ALS group at the 60-day time point (Figure 4C; day 60: 0.3512 ± 0.0743% reads in the ALS + CRD group vs. 0.1471 ± 0.0406% reads in the ALS group, *p* < 0.05), but only a slight increase at day 90-day time point (Figure 4C; day 90: 1.886 ± 0.5680% reads in the ALS + CRD group vs. 0.8807 ± 0.3429% reads in the ALS group, *p* > 0.05). These data showed that CRD might enrich the proportion cyanobacteria in the gut of ALS model mice. CRD did not lead to significant changes in the cyanobacteria levels in the WT mice at any of the three time points (Figure 4C; day 60: 0.2222 ± 0.1624% reads in the WT + CRD group vs. 0.0898 ± 0.0086% reads in the WT group, *p* > 0.05; day 90: 1.1689 ± 0.6295% reads in the WT + CRD group vs. 0.118 ± 0.0264% reads in the WT group, *p* > 0.05; day 120: 0.1892 ± 0.0579% reads in the WT + CRD group vs. 0.3335 ± 0.1574% reads in the WT group, *p* > 0.05). Moreover, no significant difference in the cyanobacteria levels was found between the ALS + CRD group and the WT + CRD group at any of the three time points (Figure 4C; day 60: 0.3512 ± 0.0743% reads in the ALS + CRD group vs. 0.2222 ± 0.1624% reads in the WT + CRD group, *p* > 0.05; day 90: 1.8862 ± 0.5680% reads in the ALS + CRD group vs. 1.1689 ± 0.6295% reads in the WT + CRD group, *p* > 0.05; day 120: 0.2222 ± 0.0973% reads in the ALS + CRD group vs. 0.1892 ± 0.0579% reads in the WT + CRD group, *p* > 0.05).

## Discussion

Accumulating evidence suggests that disturbed circadian rhythms contribute to the progression of several neurodegenerative diseases such as AD and PD, and here, we provide evidence that CRD could contribute to ALS progression as well. We found that CRD exacerbated the degeneration of MNs in the spinal ventral horn, increased the numbers of glial cells, activated the NF-κB inflammatory pathway, increased the numbers of gut cyanobacteria, and accelerated both the onset and progression of the disease in the presence of an SOD1 mutation.

Disturbed circadian rhythms are comorbid with psychiatric abnormalities including stress, anxiety, depression, schizophrenia, and suicide (34). Transgenic animal models of clock disruptions such as PER1Brdm1−/−, CRY1−/−, and CRY2−/− mutant mice displayed evident depression or anxiety-like behaviors, as well as arrhythmic feeding behavior and impaired glucose tolerance (35). Interestingly, several of the above pathological states have been observed in patients and/or animals with ALS. Persistent exposure to intense stress led to more activation of astrocytes and microglia in the spinal cord, accelerated symptoms, and shortened the median survival period in mouse models with ALS (36). Disturbed eating behaviors and metabolic dysfunction worsened disease progression and prognosis of ALS (37). Additionally, the glial immune reactions that are crucial components of the process underlying ALS were found to follow a circadian variation in the mouse SCN, and could mediate peripheral signals being sent to the central circadian clock (38). Here, astrocytic and microglial activation in the spinal cord accompanying the disruption of circadian rhythmicity may support the role of glial immune reactions in ALS.

Many immunohistochemistry studies have shown that NF-κB is activated in glia in patients with familial and sporadic ALS and in animal models of the disease (39, 40). Consistent with these findings, we found a gradual increase in the activation of the constituents of the NF-κB pathway in the spinal cord in association with disease progress in SOD1G93A ALS mice. More direct evidence indicates that inappropriate NF-κB activation is the pathogenic mechanism underlying optineurin mutation-related ALS (41, 42). The circadian clock controls many physiological changes mediated by activation of the transcription factor NF-κB (43). In our study, activation of the NF-κB pathway in the spinal cord caused by circadian rhythm disorder further confirmed that NF-κB-induced inflammation mediates the connection between abnormal circadian rhythm and neuronal death in ALS model mice. Microglia, but not astrocytes, can induce MN death *via* the classical NF-κB pathway in ALS (44). Therefore, whether the effects of the abnormal circadian rhythm were mainly mediated by microglia or astrocytes requires further investigation, as both cell types were activated in our study. More intense inflammatory reactions were also seen in WT mice exposed to the unusual circadian rhythm, although such inflammatory changes were not sufficient to cause neuronal death in WT mice. We thus conclude that circadian rhythm disorder catalyzes pathological progress in individuals with ALS due to genetic mutations, although it is not a causative factor. If this is true, then prophylactic treatments to adjust the circadian rhythm might be required for patients with familiar ALS. Although our work defines CRD as a risk factor for ALS progression, the exact mechanisms linking the circadian clock and MNs, and the subsequent upregulation of inflammatory responses requires further in-depth studies.

The gut microbiome and the SCN circadian clock may interact reciprocally in complex ways (23–25). We, therefore, hypothesize that the negative effects of CRD on the progression of ALS result from multifaceted mechanisms involving the intestinal microbiota. The recent study regarding the colonization of microbiota from patients with PD cited in the Section “Introduction” demonstrates the importance of gut microbes in PD (26). Furthermore, constipation is a common and early non-motor symptom in patients with PD that can be improved by treatment with *Lactobacillus casei* Shirota (45). However, similar studies on the effects of gut microbes in ALS are rare, although gut health-related problems, such as eating difficulties, lower physical activity, and mood disorders, are common in patients with ALS (46). The use of 16S rRNA sequencing has improved the correlative analysis of gut microflora and disease. Recently, shifts in the intestinal microbiome and aberrant intestinal homeostasis were reported to be associated with ALS pathophysiology in SOD1G93A mice following 454 pyrosequencing (47). The oral administration of butyrate restored intestinal microbiota homeostasis, and thus delayed disease onset and progression in the SOD1G93A mice (47).

The prevalence of ALS is greater in areas with abundant cyanobacteria or high levels of BMAA (48, 49). We found a remarkable increase of the cyanobacteria abundance at day 90, shortly after disease onset in the ALS model mice, which hints a link between intestinal cyanobacteria and the onset and progression of ALS. Gut microflora are vulnerable to the changes of host diet, circadian clock, genotype, age, sex, medicine, and other conditions (50). In this study, the relative abundance of cyanobacteria was enriched by CRD in the ALS model mice at the day 60. However, there were no statistically significant differences in intestinal cyanobacteria in the terminal stages among the four groups. We suspect that this may be due to extremely disordered floral changes due to many different factors, such as an inability to eat or move at the end stages of the disease in ALS mice. Thus, it is tempting to conclude that CRD gradually desynchronizes the intestinal activity of cyanobacteria in the early stage of ALS progression. Cyanobacteria generate BMAA, which has been shown to be neurotoxic and correlated with the higher incidence of ALS-related neurodegenerative disease in Guam (48). Additionally, a recent study found that neonatal mice treated with BMAA had long-term learning impairments (51). Another study analyzing the gut in SOD1G93A mice demonstrated deficits in the tight junction protein zonula occludens-1 leading to increased gut permeability. The above report thus highlights a putative mechanism by which neurotoxins can move from the gut to the brain (52). Our results provide evidence that circadian rhythm disorders promote ALS disease onset and progression, possibly by increasing the abundance of gut cyanobacteria in the mice. However, assessments of the levels of BMAA and other cyanobacterial neurotoxins, such as saxitoxin and anatoxin (53–55), will be necessary in determining the mechanisms underlying the effects of gut cyanobacteria on disease onset and progression in ALS.

## Ethics Statement

This study was carried out in accordance with the institutional recommendations for the care and use of experimental animals of the research and ethics committee. The protocol was approved by the Animal Ethical Committee at Sun Yat-Sen University, China.

## Author Contributions

ZH, HS, and XY contributed to the conception and design of the study; ZH, QL, YP and SL organized the database; JD, YX and WC performed the statistical analysis; ZH, HS and XY wrote the first draft of the manuscript; QL and ZP wrote sections of the manuscript. All authors contributed to manuscript revision, read and approved the submitted final version.

## Conflict of Interest Statement

The authors declare that the research was conducted in the absence of any commercial or financial relationships that could be construed as a potential conflict of interest.
